# Cognitive impact after short-term exposure to different proton pump inhibitors: assessment using CANTAB software

**DOI:** 10.1186/s13195-015-0164-8

**Published:** 2015-12-27

**Authors:** Sanjida Akter, Md. Rajib Hassan, Mohammad Shahriar, Nahia Akter, Md. Golam Abbas, Mohiuddin Ahmed Bhuiyan

**Affiliations:** Department of Pharmacy, School of Medicine, University of Asia Pacific, House no. 73, Road no. 5A, Dhanmondi, Dhaka 1209 Bangladesh; Department of Molecular Neuroscience and Integrative Physiology, Graduate School of Medical Science, Kanazawa University, Kanazawa, Ishikawa Japan

## Abstract

**Introduction:**

Studies have shown that proton pump inhibitors (PPIs) increase the brain burden of amyloid-beta (Aβ) and also create vitamin B_12_ deficiency. However, these two phenomena have deleterious effect on cognition and Alzheimer’s disease (AD). Since the use of PPIs has increased tremendously for the last few years, it is of great public health importance to investigate the cognitive impact of PPIs. Hence, the purpose of this study was to investigate the degree of neuropsychological association of each PPI with different cognitive functions.

**Methods:**

Sixty volunteers of either gender were recruited and divided randomly into six groups: five test groups for five classes of PPIs and one control group. All the groups participated in the five computerized neuropsychological tests (nine subtests) of the Cambridge Neuropsychological Test Automated Battery twice: at the beginning of the study and 7 days thereafter.

**Results:**

We found statistically and clinically significant impairment in visual memory, attention, executive function, and working and planning function. One-way analysis of variance findings showed that all PPIs had a similar negative impact on cognition. However, paired-samples *t* tests indicated that omeprazole showed significant (*p* < 0.05) results in seven subtests; lansoprazole and pantoprazole showed significant results in five subtests; and rabeprazole showed significant results in four subtests. Among five classes of PPIs, esomeprazole showed comparatively less impact on cognitive function with significant results in three subtests.

**Conclusions:**

The present study reveals for the first time that different PPIs have varying degrees of influence on different cognitive domains and have associations with AD. These findings should be considered when balancing the risks and benefits of prescribing these medications. A study done for a longer period of time with a larger sample size might yield better results.

## Introduction

Proton pump inhibitors (PPIs) are one of the most frequently prescribed classes of drugs in the world due to their few immediate and tangible side effects [1]. Major indications for PPI therapy include peptic ulcer disease, gastroesophageal reflux disease, refractory or resistant erosive esophagitis, Zollinger-Ellison syndrome, chronic nonsteroidal anti-inflammatory drug use, and treatment of *Helicobacter pylori* [2]. All the PPIs act by forming irreversible disulfide bonding with the cysteine residue of the hydrogen potassium adenosine triphosphatase (ATPase, proton pump), thus inhibiting the secession of gastric acid from the parietal cell [3, 4].

The strong evidence supporting the superior safety and efficacy of PPIs has made PPIs the mainstay of therapy compared with other antisecretory agents used [5]. With annual U.S. sales of $13.9 billion, they are the third most widely sold drug class in the United States [2]. From 2009 to 2013, the number of prescriptions increased from 146 million to 164 million, acquiring eighth position in the list of the total prescription share of top therapeutic classes [6]. However, studies have shown that 25–70 % of patients taking these drugs have no appropriate indication [7]. This is because the PPIs are constantly being overprescribed in both primary and secondary care globally [8–12]. There is evidence that elderly patients are unnecessarily prescribed PPIs at the time of hospital admission as “gastroprotection” with the sole aim of avoiding potential legal prosecution of physicians in charge for ignoring medical care [13, 14]. Such overprescribing and overuse of PPIs raises concern about the aftermath of PPI-induced health hazards. In general, PPIs are considered safe, with minor adverse effects ranging from approximately 1 to 3 % [15, 16]. However, numerous adverse effects, particularly those associated with long-term use, have been reported [2]. Although many authors have focused on common side effects such as headache, nausea, diarrhea, dizziness, and rash, some scientists very recently have shown that long-term PPI therapy has an exacerbated effect on human cognition. In a longitudinal, multicenter cohort study involving primary care elderly patients, Haenisch et al. [17] showed that patients receiving PPI medication had a significantly increased risk of any dementia as well as Alzheimer’s disease (AD).

AD is a progressive neurodegenerative disease characterized by dementia associated with impaired memory, language, and general intellectual activities [18]. One of the prime neuropathological hallmarks of AD is the extracellular deposition of amyloid-beta (Aβ) peptides in the brain [19, 20].

In 2013, Badiola et al. [21] explored for the first time the effect of lansoprazole and other PPIs on Aβ production by using cellular and animal models. They suggested that PPIs modulate β-site amyloid precursor protein-cleaving enzyme 1 and γ-secretase, two protease enzymes responsible for sequential cleavage of amyloid precursor protein, resulting in formation of Aβ. It has also been shown that PPIs can cross the blood–brain barrier and block the vacuolar-type ATPase proton pumps (V-ATPases) Blocking of V-ATPases results in increased pH of microglial lysosomes, leading to decreased degradation of Aβ by microglial phagocytosis [22, 23]. Finally, accumulation of Aβ oligomer forms insoluble plaques in the brain that potentiate the formation of cytotoxic inflammatory cytokines and reactive oxygen species, which may indirectly cause neurodegeneration and hamper brain function [24].

These findings are particularly important for elderly recipients of chronic PPI therapy because long-term PPI therapy may potentiate AD progression or aggravate AD symptoms in these patients. Furthermore, long-term PPI therapy for elderly patients may precipitate vitamin B_12_ deficiency due to malabsorption of protein-bound vitamin B_12_ [25–27]. Poor vitamin B_12_ status has been linked with cognitive decline [28–30] and AD [28, 31, 32]. The probable etiologies of this association include atrophy of the cerebral cortex and white matter damage in the central nervous system due to demyelination [33], impaired DNA synthesis, and accumulation of neurotoxic total homocysteine and/or methylmalonic acid [28, 34, 35].

However, until recently, little evidence existed about the impact of PPIs on cognition. Haenisch et al. [17] reported an inverse relationship of PPI use with cognition. In their epidemiologic study, they showed only an overall rise of dementia and AD risk for patients with chronic PPI therapy and did not mention anything about which cognitive functions were impaired by specific PPIs. As a result, further clinical investigation is required to draw inferences regarding the effect of each PPI on different cognitive functions.

Rather than using written questionnaires in this study, we used Cambridge Neuropsychological Test Automated Battery (CANTAB) software (Cambridge Cognition, Cambridge, UK), which is a semiautomated computer program that employs touch screen technology used in numerous neurocognitive studies over the last two decades [36]. So far, researchers at 700 institutions have used Cambridge Cognition’s CANTAB technology in 150 clinical trials to assess cerebral diseases such as Parkinson’s disease, AD, schizophrenia, and depression. CANTAB’s 25 neuropsychological tests are divided into 7 main groups: screening tests, visual memory tests, executive function, working memory and planning tests, attention tests, semantic/verbal memory tests, decision-making and response control tests, and social cognition and other tests [37]. In this study, we used the CANTAB Dementia Battery, which comprises five tests [Motor Screening Test (MOT), Paired Associates Learning (PAL), reaction time (RTI), rapid visual information processing (RVP), and spatial working memory (SWM)] designed to detect most subtle changes in cognition. This battery is often used in clinical research to identify preclinical AD [38, 39].

The goal of this study was to investigate the effect of different PPIs (lansoprazole, omeprazole, pantoprazole, rabeprazole, and esomeprazole) on visual memory, executive function, working memory, planning and strategy development, speed of response, and sustained attention in healthy Bangladeshi individuals and to establish some correlations between each agent and these functions when administered in a maximal daily dose.

## Methods

### Participants

In the present study, a total of 60 healthy volunteers, including both sexes, were recruited randomly. The participants’ mean ages were 23 years for men and 21 years for women (overall range 20–26 years). Written informed consent was obtained from the volunteers before they entered study, and they were introduced with a complete set of medical health questions for evaluation of their health conditions. Participants recruited were in normal physical health and without any cardiac, gastric, renal, or hepatic disease. None of them had any previous history of psychological limitations, and none had consumed PPIs within 3 months before entering the study. They were advised to follow a standard diet during the study period and to avoid alcoholic beverages, nicotine, or any type of medication for at least 48 hours before each experimental session. They also were advised not to consume caffeinated beverages for at least 24 hours before testing. No financial compensation was allocated for their participation.

### Study design and procedures

**Fig. 1:**
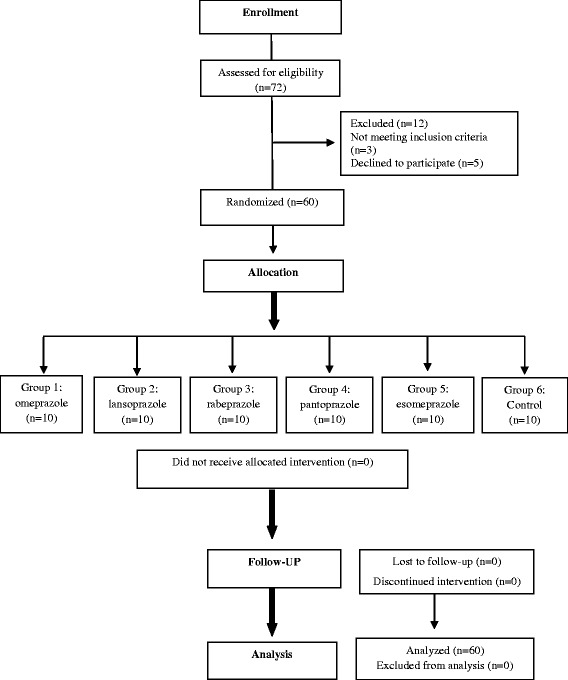
Consort flow diagram

The study was conducted over the course of 1 week. The volunteers were randomly divided into one of five treatment groups (to receive one of five classes of PPIs) or to a control group. Each group consisted of ten volunteers. The members of the treatment groups were receiving a maximum daily dose of one of the respective PPIs (Fig. 1). Participants in group 1 received omeprazole 40 mg/day; group 2 received lansoprazole 30 mg/day, group 3 received rabeprazole 20 mg/day; group 4 received pantoprazole 40 mg/day; and group 5 received esomeprazole 40 mg/day. The control group (group 6) received a placebo capsule. The placebo was a husk of isabgol (psyllium seed husk) within a hard gelatin capsule shell (size 0).

The volunteers in the present study were assessed with the CANTAB for all the parameters measured at baseline and at 7 days of treatment. The instructions for the tests were explained to the volunteers before initiation of the study. All the participants were kept blinded about whether they were taking a PPI or placebo, and the group allocation was revealed only after the assessment of the last subject. All the participants were instructed to call the study center in case of any adverse effect during the study. They had the opportunity to withdraw from the study at any time. The volunteers were contacted at defined intervals to ensure that they were taking the dose regularly.

### Assessments

The volunteers were asked to sit for a series of five computerized neuropsychological tests (the CANTAB Dementia Battery) mentioned in the CANTAB manual protocols.

#### Screening/familiarization test

##### Motor Screening Test

This test is common to all of the CANTAB batteries and is performed at the beginning of a test session. The purpose of the test is to relax the subject and to introduce the subject to the computer and touch screen. The test simultaneously screens for difficulties with vision, movement, and comprehension and ascertains whether the subject can follow simple instructions. A series of flashing crosses is shown in different locations on the screen, and the subject is instructed to point to each cross as soon as it appears, using the forefinger of the dominant hand.

The following are the outcome measures used in the MOT:

- *MOT mean latency*: Measures how quickly the subject touches the cross after it appears
- *MOT mean error*: Measures how accurately the subject touches the cross by measuring the mean distance between the center of the cross and the location the subject touches on the screen, for the ten crosses presented to which the subject correctly responds

#### Visual memory test

##### Paired Associates Learning

PAL tests visual memory and new learning. This test is sensitive primarily to changes in the temporal and frontal lobes. In this test, initially six boxes are exhibited on the screen, and they are opened in a randomized order to reveal the contents. One or more of the boxes will contain a pattern. Each pattern is then displayed in the middle of the screen, one at a time, and the participant must identify the box containing the particular pattern. When the participant gets all the locations correct, he or she proceeds to the next stage, which includes eight boxes; otherwise, the test terminates. The test has an increasing level of difficulty ranging from two to eight patterns to be remembered [40].

The following are the outcome measures used in the PAL test:

- *PAL total errors adjusted*: The total number of errors made in all stages, along with an adjustment for each stage not attempted owing to previous failure
- *PAL total errors (six shapes, adjusted)*: The total number of errors in the eight-stage pattern, with an adjustment made for subjects who have not reached this stage

#### Attention tests

##### Reaction time

The RTI test is designed to assess the speed of the subject’s motor and mental responses to a visual target that appears on the screen, where the stimulus is either predictable (simple reaction time) or unpredictable (choice reaction time). The five successive stages of the task require increasingly complex chains of responses [39]. A yellow spot may appear either in one location or in one of five locations, and the participant must respond sometimes by using the press pad, sometimes by touching the screen, and sometimes both.

The following are the outcome measures used in the RTI test:

- *RTI five-choice reaction time*: Measures the subject’s response latency for releasing the press pad in response to the onset of a stimulus in one of five locations
- *RTI five-choice movement time*: Time taken to touch the stimulus (on the touch screen) after the press pad has been released

##### Rapid visual information processing

This test is sensitive to the activity of the frontoparietal lobe and measures the subject’s visual sustained attention and working memory [41]. The participant has to depress a press pad upon recognizing a sequence of digits from among a pseudo-random order of digits that appears in the center of the screen at a rate of 100 digits per minute.

The following outcome measure is used in the RVP test:

- *RVP A′*: The probability of detecting the target sequence

#### Executive function, working memory, and planning test

##### Spatial working memory

The SWM test measures the retention and manipulation of visuospatial information and also assesses heuristic strategy [42]. The test is a sensitive measure of executive dysfunction of the frontal lobe [43]. In this test, the participant must search some colored square boxes on the screen to find a blue “token” and use it to fill an empty bar on the right-hand side of the screen. The number of boxes is gradually increased from three to eight. To discourage the use of stereotyped search strategies, the colors and positions of the boxes are changed from trial to trial.

The following are the outcome measures used in the SWM test:

- *SWM between errors*: Number of times the volunteer visits the box in which the blue token has already been found
- *SWM strategy*: Number of times the participant begins a search with the same box for six to eight box problems

### Statistical analysis

Statistical analyses were performed using IBM SPSS for Windows version 20.0 software (IBM, Armonk, NY, USA). All the outcome measures were analyzed for normal distribution using the Shapiro-Wilks test and were found to be normal. Levene’s test was used for the test of the equality of variances; in cases of significant outcomes, it was followed by the Welch test.

When the parametric assumptions were satisfied, one-way analysis of variance (ANOVA) was carried out to investigate possible differences in outcomes among the five drug groups. No post hoc tests were performed, as we found no significant differences among the drug groups in ANOVA. Differences in mean performance within groups from baseline to posttreatment were analyzed by using a parametric paired-samples *t* test. However, an independent samples *t* test was also employed to analyze the outcome between each treatment group and the control group for the second-session data. All data were measured at 95 % confidence intervals (CIs), and the threshold for statistical significance was set at *p* < 0.05.

The effect size of scores was analyzed by Cohen’s *d* value, and the CI was also calculated to observe not only statistical significance but also the practical and clinical significance of the findings.

### Ethics statement

All procedures in this study were approved by the Clinical Research Ethics Committee, School of Medicine, University of Asia Pacific, Dhaka, Bangladesh, and were carried out in accordance with the Declaration of Helsinki of 1975, as revised in 2008.

## Results

### Motor Screening Test

We subjected all the participants to the MOT to familiarize them with the CANTAB interface. This gave us a general idea about whether the participants had any sensorimotor or other difficulties that may have hampered the collection of valid data for the subsequent tasks. We found that the participants had a significant reduction in capacity to follow the instructions, as well as an increase in time needed to complete the respective tasks accurately.

We found significant (*p* < 0.05) increases in mean error from baseline to 7 days of treatment in the omeprazole (*p =* 0.038) and esomeprazole (*p =* 0.038) groups, with a large effect size. The lansoprazole and pantoprazole groups both showed significant increases (*p =* 0.014 and *p =* 0.009, respectively) in mean latency time, with a large effect size (Table 1).

The posttreatment effect of PPIs and placebo (control) showed no significant changes, except for the esomeprazole and pantoprazole groups (*p =* 0.010 and *p =* 0.045, respectively), with a large effect size. However, one-way ANOVA showed no significant differences in mean latency score [*p =* 0.828, *F*(4, 45) = 0.371] or mean error score [*p =* 0.731, *F*(4, 45) = 0.507] among the groups taking PPIs (Table 6).

### Paired Associates Learning

The PAL test evaluates visual memory and learning of the participants. We discovered that participants experienced difficulties in choosing the correct location of the pattern and needed more trials to choose the correct location after consuming PPIs.

A paired-samples *t* test showed that omeprazole, lansoprazole, and pantoprazole significantly (*p <* 0.05) increased mean values within each group before treatment and after treatment. The omeprazole group showed significant increases in PAL total error adjusted score (*p =* 0.013) and PAL total error six-shapes adjusted score (*p =* 0.023) with 95 % CIs, with a large effect size (Table 2). Likewise, the lansoprazole and pantoprazole groups also showed significantly different error scores (*p =* 0.012 and 0.046, respectively) among the groups before and after treatment. Although rabeprazole and esomeprazole increased the mean value, the differences were not statistically significant.

When we compared all the PPI groups with the control group, we found that only participants in the omeprazole and lansoprazole groups scored significantly (*p =* 0.042 and 0.034, respectively). One-way ANOVA of the five PPI groups indicated no significant differences among them in increasing the PAL total error adjusted score [*p =* 0.580, *F*(4, 45) = 0.724] or the PAL total error six-shapes adjusted score [*p =* 0.315, *F*(4, 45) = 1.222] (Table 6).

### Reaction time

This task is designed to evaluate motor and mental response speeds such as reaction time, movement time, and reaction accuracy. Our data indicate that participants receiving PPIs took more time to react upon seeing a visual stimulus.

RTI movement time score increased significantly (*p <* 0.05) from baseline to posttreatment with borderline significance for omeprazole (*p =* 0.021, 95 % CI −215.510 to −4.090), pantoprazole (*p =* 0.008, 95 % CI −226.400 to −28.600), lansoprazole (*p =* 0.033), and rabeprazole (*p =* 0.030). The large Cohen’s *d* values for these four treatment groups indicate the magnitude of differences between the baseline and posttreatment data (Table 3). However, the data for the esomeprazole group remained nonsignificant (*p >* 0.05). Despite our finding of a significant rise in RTI movement time in almost all the treatment groups (except esomeprazole), none of the drugs increased RTI reaction time significantly (*p >* 0.05).

**Table 1:** Screening or familiarization test

Motor Screening Test (MOT)									
Drug	Name of task	Mean ± SEM		*p1* value	95 % CI-D (Lower, Upper)	Cohen’s *d* for *p1* Value	*p2* Value	95 % CI-D (Lower, Upper)	Cohen’s *d* for *p2* Value
		Baseline	After 7 Days						
OME	MOT M latency	784.300 ± 48.095	849.200 ± 80.201	0.206	−235.741, 105.941	0.573	0.292	−143.601, 244.661	0.305
	MOT M error	7.400 ± 0.427	9.600 ± 1.166	0.038*	−4.696, 0.296	1.329^**ǂ**^	0.187	−1.577, 3.997	0.429
LAN	MOT M latency	744.400 ± 24.413	865.700 ± 58.618	0.014*	−226.980, -15.620	1.731^**ǂ**^	0.181	−83.723, 217.563	0.439
	MOT M error	8.600 ± 0.686	9.600 ± 0.968	0.171	−3.262, 1.262	0.667	0.101	−0.885, 3.813	0.702
RAB	MOT M latency	890.000 ± 53.938	883.900 ± 48.187	0.470	−171.647, 184.847	0.051	0.098	−48.222, 218.502	0.632
	MOT M error	7.000 ± 0.615	8.500 ± 1.276	0.166	−4.808, 1.808	0.684	0.451	−2.849, 3.198	0.071
PAN	MOT M latency	768.100 ± 40.731	847.900 ± 36.554	0.009**	−143.281, -16.319	1.896^**ǂ**^	0.191	−66.545, 165.045	0.421
	MOT M error	9.700 ± 0.870	9.900 ± 0.767	0.405	−2.041, 1.641	0.164	0.045*	−0.283, 3.537	0.843^**ǂ**^
ESO	MOT M latency	696.300 ± 52.443	788.700 ± 60.783	0.096	−241.107, 56.307	0.937^**ǂ**^	0.446	−164.470,144.343	0.065
	MOT M error	9.100 ± 0.722	10.300 ± 0.597	0.044*	−2.623, 0.223	1.272^**ǂ**^	0.010**	0.352, 3.748	1.196^**ǂ**^
CON	MOT M latency	853.700±53.702	798.700 ± 41.260	0.088	−29.868, 139.868	N/A			
	MOT M error	9.500 ± 0.687	8.300 ± 0.539	0.073	−0.513, 2.913	N/A			

Values are expressed as mean ± SEM (n=10), M=Mean, 95 % CI-D=95 % Confidence Interval of the Difference (lower, upper), *p1*= *p* value found after Paired-Samples *t-*test within volunteers (five test groups) before and after consuming drugs and placebo (one control group) respectively with degree of freedom (df)=9; *p2* = *p* value found after Independent-Samples *t-*test between each five test groups with control group having degree of freedom (df)=18. * and ** indicate statistically significant at alpha (<0.05) & (<0.01) respectively. ^**ǂ**^ indicates large effect size in Cohen’s *d* value

Among the drug and control groups, we found significant increases in movement time for the groups receiving omeprazole (*p =* 0.006), rabeprazole (*p =* 0.023), and pantoprazole (*p =* 0.007), with significant 95 % CI values and large effect sizes. Interestingly, the reaction time for the treatment group taking rabeprazole decreased significantly (*p =* 0.017, 95 % CI = −169.820 to −7.443) compared with the control group. However, all the treatment groups showed similar increases in both reaction time [*p =* 0.183, *F*(4, 45) = 1.629] and movement time [*p =* 0.458, *F*(4, 45) = 0.925] (Table 6).

**Table 2:** Memory test

Paired Associates Learning (PAL)									
Drug	Name of task	Mean ± SEM		*p1* value	95 % CI-D (Lower, Upper)	Cohen’s *d* for *p1* Value	*p2* Value	95 % CI-D (Lower, Upper)	Cohen’s *d* for *p2* Value
		Baseline	After 7 Days						
OME	PAL TE (adjusted)	5.300 ± 1.212	9.900 ± 2.331	0.013*	−8.521, -0.679	1.769^**ǂ**^	0.207	−4.098, 9.498	0.393
	PAL TE(6 shapes, adjusted)	1.800 ± 0.800	3.700 ± 0.955	0.023*	−3.761, -0.039	1.540^**ǂ**^	0.042*	−0.310, 4.510	0.863^**ǂ**^
LAN	PAL TE(adjusted)	8.400 ± 1.973	14.400 ±6.143	0.151	−18.422, 6.422	0.729	0.142	−6.541, 20.941	0.519
	PAL TE(6 shapes, adjusted)	1.600 ± 0.670	5.100 ± 1.696	0.012*	−6.445, -0.555	1.793^**ǂ**^	0.034*	−0.305, 7.035	0.911^**ǂ**^
RAB	PAL TE (adjusted)	8.300 ± 1.739	8.800 ± 2.886	0.448	−8.919, 7.919	0.089	0.333	−6.081, 9.281	0.206
	PAL TE(6 shapes, adjusted)	1.400 ± 0.670	1.900 ± 1.048	0.361	−3.959, 2.595	0.243	0.404	−2.275, 2.875	0.115
PAN	PAL TE(adjusted)	4.000 ± 1.374	6.200 ± 1.988	0.046*	−4.851, 0.451	1.251^**ǂ**^	0.371	−7.299, 5.299	0.157
	PAL TE(6 shapes, adjusted)	1.500 ± 0.749	2.700 ± 1.334	0.180	−4.017, 1.617	0.643	0.233	−2.004, 4.204	0.351
ESO	PAL TE (adjusted)	10.200±1.618	9.300 ± 2.357	0.364	−4.767, 6.567	0.239	0.263	−4.739, 8.939	0.304
	PAL TE(6 shapes, adjusted)	2.400 ± 0.562	2.100 ± 0.674	0.388	−2.013, 2.613	0.195	0.298	−1.447, 2.447	0.255
CON	PAL TE(adjusted)	8.300 ± 2.530	7.200 ± 2.245	0.242	−2.313, 4.513	N/A			
	PAL TE(6 shapes, adjusted)	2.500 ± 1.138	1.600 ± 0.636	0.194	−1.348, 3.148	N/A			

Values are expressed as mean ± SEM (n=10), TE=Total errors, 95 % CI-D=95 % Confidence Interval of the Difference (lower, upper), *p1*= *p* value found after Paired-Samples *t-*test within volunteers (five test groups) before and after consuming drugs and placebo (one control group) respectively with degree of freedom (df)=9; *p2*= *p* value found after Independent-Samples *t-*test between each five test groups with control group having degree of freedom (df)=18.* indicates statistically significant at alpha (<0.05). ^**ǂ**^ indicates large effect size in Cohen’s *d* value

**Table 3:** Attention test (speed of response & movement)

Reaction Time (RTI)									
Drug	Name of task	Mean ± SEM		*p1* value	95 % CI-D (Lower, Upper)	Cohen’s *d* for *p1* Value	*p2* Value	95 % CI-D (Lower, Upper)	Cohen’s *d* for *p2* Value
		Baseline	After 7 Days						
OME	RTI FC movement time	440.800 ± 70.397	550.600 ±33.557	0.021*	−215.510, -4.090	1.567^**ǂ**^	0.006**	43.597, 311.193	1.313^**ǂ**^
	RTI FC reaction time	401.600 ± 24.419	401.700 ±47.429	0.499	−84.483, 84.283	0.002	0.228	−160.450,75.203	0.358
LAN	RTI FC movement time	429.600 ± 42.988	494.200 ±62.281	0.033*	−134.633, 5.433	1.391^**ǂ**^	0.080	−52.458, 294.283	0.691
	RTI FC reaction time	411.500 ± 34.816	427.100 ±19.626	0.337	−96.876, 65.676	0.289	0.319	−92.282, 58.072	0.225
RAB	RTI FC movement time	441.400 ± 52.311	541.700 ±56.709	0.030*	−206.318, 5.718	1.427^**ǂ**^	0.023*	3.586, 332.896	1.012^**ǂ**^
	RTI FC reaction time	350.900 ± 18.929	355.500 ±24.407	0.389	−40.606, 31.406	0.193	0.017*	−169.820, -7.443	1.081^**ǂ**^
PAN	RTI FC movement time	431.400 ± 31.271	558.900 ±40.799	0.008**	−226.400, -28.600	1.944^**ǂ**^	0.007**	43.011, 327.913	1.289^**ǂ**^
	RTI FC reaction time	438.500 ± 29.655	459.000 ±28.355	0.275	−95.345, 54.345	0.413	0.361	−71.730, 101.519	0.170
ESO	RTI FC movement time	10.200 ± 1.618	9.300 ± 2.357	0.248	−178.053, 93.053	0.473	0.187	−91.254, 230.490	0.428
	RTI FC reaction time	2.400 ± 0.562	2.100 ± 0.674	0.297	−127.810, 77.610	0.369	0.303	−99.234, 165.251	0.247
CON	RTI FC movement time	415.100 ± 52.731	371.300 ±54.126	0.182	−57.137, 140.737	N/A			
	RTI FC reaction time	466.600 ± 29.218	444.000 ±29.954	0.128	−19.658, 64.858	N/A			

Values are expressed as mean ± SEM (n=10), FC=Five-Choice, 95 % CI-D=95 % Confidence Interval of the Difference (lower, upper), *p1*= *p* value found after Paired-Samples *t-*test within volunteers (five test groups) before and after consuming drugs and placebo (one control group) respectively with degree of freedom (df)=9; *p2*= *p* value found after Independent-Samples *t-*test between each five test groups with control group having degree of freedom (df)=18. * and ** indicate statistically significant at alpha (<0.05) & (<0.01) respectively. ^**ǂ**^ indicates large effect size in Cohen’s *d* value

### Rapid visual information processing

RVP is a test of sustained attention or vigilance that requires working memory. It is also a sensitive measure of general information-processing performance. RVP A′ is a subtest of RVP that measures how well a subject can detect target sequences.

Across all the treatment groups, there were statistically significant decreases in RVP A′ scores. According to a paired-samples *t* test, participants in the omeprazole, lansoprazole, rabeprazole, pantoprazole, and esomeprazole groups showed significant decreases in RVP A′ scores (*p =* 0.030, *p =* 0.043, *p =* 0.037, *p =* 0.029, and *p =* 0.032, respectively) (Table 4). In addition, large Cohen’s *d* values for these groups indicated the practical significance of the test scores. In contrast to within-group mean variations, no effects were found among the treatment groups and the control group (*p >* 0.05). Participants receiving drug treatment showed similar decreases in RVP A′ scores, as suggested by ANOVA among the treatment groups [*p =* 0.796, *F*(4, 45) = 0.417] (Table 6).

**Table 4:** Attention test (visual sustained attention)

Rapid Visual Information Processing (RVP)									
Drug	Name of task	Mean ± SEM		*p1* value	95 % CI-D (Lower, Upper)	Cohen’s *d* for *p1* Value	*p2* Value	95 % CI-D (Lower, Upper)	Cohen’s *d* for *p2* Value
		Baseline	After 7 Days						
OME	RVP A'	0.940 ± 0.014	0.920 ± 0.015	0.030*	−0.001, 0.050	1.428^**ǂ**^	0.492	−0.045, 0.044	0.009 (S)
LAN	RVP A'	0.920 ± 0.013	0.900 ± 0.009	0.043*	−0.004, 0.044	1.281^**ǂ**^	0.251	−0.049, 0.025	0.322 (S)
RAB	RVP A'	0.934 ± 0.013	0.900 ± 0.016	0.037*	−0.004, 0.072	1.343^**ǂ**^	0.248	−0.062, 0.031	0.327 (S)
PAN	RVP A'	0.931 ± 0.010	0.900 ± 0.016	0.029*	−0.001, 0.063	1.445^**ǂ**^	0.248	−0.063, 0.031	0.328 (S)
ESO	RVP A'	0.940 ± 0.016	0.918 ± 0.010	0.032*	−0.002, 0.046	1.398^**ǂ**^	0.441	−0.035, 0.040	0.071 (S)
CON	RVP A'	0.901 ± 0.013	0.915 ± 0.015	0.217	−0.054, 0.025	N/A			

Values are expressed as mean ± SEM (n=10), 95 % CI-D=95 % Confidence Interval of the Difference (lower, upper), *p1*= *p* value found after Paired-Samples *t-*test within volunteers (five test groups) before and after consuming drugs and placebo (one control group) respectively with degree of freedom (df)=9; *p2*= *p* value found after Independent-Samples *t-*test between each five test groups with control group having degree of freedom (df)=18 . * indicates statistically significant at alpha (<0.05). ^**ǂ**^ indicates large effect size in Cohen’s *d* value

### Spatial Working Memory

The SWM test assesses the retention and manipulation of visuospatial information into working memory. Of the five cognitive tests we carried out to assess the rate and extent of cognitive function, we found comparatively profound results in the SWM test compared with baseline scores (significant *p* value and 95 % CI, and large effect size) among the five groups receiving PPIs (Table 5). Omeprazole and rabeprazole significantly increased SWM between errors scores (*p =* 0.002 and 0.016, respectively) and SWM strategy scores (*p =* 0.012 and 0.022, respectively). Participants in the lansoprazole, pantoprazole, and esomeprazole groups made significantly more errors in the SWM strategy test (*p =* 0.001, *p =* 0.010, and *p =* 0.035, respectively). However, no significance was found in the SWM between error scores among these groups. Interestingly, none of the treatment groups’ scores differed significantly from the control group’s.

**Table 5:** Executive function, working memory and planning test

Spatial Working Memory (SWM)									
Drug	Name of task	Mean ± SEM		*p1* value	95 % CI-D (Lower, Upper)	Cohen’s *d* for *p1* Value	*p2* Value	95 % CI-D (Lower, Upper)	Cohen’s *d* for *p2* Value
		Baseline	After 7 Days						
OME	SWM BE	15.800 ± 3.123	29.500 ± 4.465	0.002**	−21.794, -5.606	2.553^**ǂ**^	0.111	−6.717, 26.917	0.595
	SWM Strategy	32.500 ± 0.703	34.900 ± 0.924	0.012*	−4.401, -0.399	1.809^**ǂ**^	0.385	−4.038, 3.038	0.140
LAN	SWM BE	29.100 ± 5.664	34.900 ± 6.781	0.153	−17.906, 6.306	0.723	0.060	−4.444, 35.444	0.769
	SWM Strategy	34.700 ± 0.895	37.700 ± 0.967	0.001***	−4.720, -1.280	2.631^**ǂ**^	0.097	−1.288, 5.888	0.635
RAB	SWM BE	22.500 ± 5.119	32.200 ± 7.360	0.016*	−18.429, -0.971	1.676^**ǂ**^	0.106	−8.031, 33.631	0.608
	SWM Strategy	34.100 ± 1.215	36.500 ± 1.607	0.022*	−4.717, -0.083	1.562^**ǂ**^	0.306	−3.389, 5.589	0.243
PAN	SWM BE	21.600 ± 4.766	31.500 ± 6.114	0.095	−25.730, 5.930	0.943^**ǂ**^	0.098	−6.869, 31.069	0.632
	SWM Strategy	33.400 ± 1.056	36.100 ± 1.027	0.010**	−4.861, -0.539	1.885^**ǂ**^	0.346	−2.961, 4.361	0.189
ESO	SWM BE	24.900 ± 4.451	28.800 ± 6.141	0.244	−16.126, 8.326	0.481	0.165	−9.606, 28.406	0.489
	SWM Strategy	33.500 ± 0.719	35.200 ± 0.940	0.035*	−3.579, 0.179	1.365^**ǂ**^	0.453	−3.757, 3.357	0.055
CON	SWM BE	19.400 ± 4.492	19.400 ± 6.644	0.500	−11.406, 11.406	N/A			
	SWM Strategy	34.400 ± 1.046	35.400 ± 1.408	0.118	−2.784, 0.784	N/A			

Values are expressed as mean ± SEM (n=10), BE=Between Errors, 95 % CI-D=95 % Confidence Interval of the Difference (lower, upper), *p1*= *p* value found after Paired-Samples *t-*test within volunteers (five test groups) before and after consuming drugs and placebo (one control group) respectively with degree of freedom (df)=9; *p2*= *p* value found after Independent-Samples *t-*test between each five test groups with control group having degree of freedom (df)=18. *, ** and *** indicate statistically significant at alpha (<0.05), (<0.01) & (<0.001) respectively. ^**ǂ**^ indicates large effect size in Cohen’s *d* value

Finally, one-way ANOVA carried out over the five treatment groups did not reveal any significant differences for SWM between error [*p =* 0.962, *F*(4,45) = 0.149] and SWM strategy [*p =* 0.426, *F*(4, 45) = 0.984] scores within the PPI groups (Table 6).

## Discussion

The rationale for conducting this study followed from the previously reported finding that lansoprazole and other PPIs increase Aβ, not only in cell cultures but also in mouse brain [21]. Another reason was that during AD accumulation of Aβ in the parietal cortex is associated with impaired visuospatial skill, executive function, and attention. Furthermore, impaired visual and verbal memory are due to accumulation of Aβ in the medial temporal lobe of the human brain [44–47].

Another aspect of how PPI consumption influences cognitive function is rather elusive. It has been suggested that chronic PPI consumption results in malabsorption of vitamin B_12_, leading to cognitive decline [17, 27, 28]. Although this phenomenon is unlikely to occur in healthy people who follow a normal diet, it might be of significant importance for elderly patients who are relatively malnourished and receiving chronic PPI therapy [48].

To assess cognitive impairment, we used the CANTAB Dementia Battery (MOT, PAL, RTI, RVP, and SWM), which is well documented to accurately assess amyloid-related cognitive decline and measure the severity of impairment in patients with prodromal AD [42, 49, 50]. Different parts of the brain control the performance of different tasks. The performance of PAL relies on the function of the temporal and frontal lobes of the cerebral cortex, whereas SWM performance relies on frontal lobe function [34, 47, 51]. RVP measures sustained attention, which is controlled by frontoparietal cortical areas [52]. RTI assesses reaction time and movement time, which are controlled by a combination of different brain areas, though scientists found that the precuneus, the posteromedial portion of the parietal lobe, plays a major role in controlling reaction time [53].

Several novel and important findings arose from the present study. In agreement with our hypothesis, significant differences were found in test scores within treatment groups before and after treatment and also between the treatment groups and the control group. These findings reveal that consumption of different PPIs may have varying degrees of influence on cognitive capacity.

**Table 6:** Levene, Welch and one way ANOVA output

Tests Used	Feature of Test	Sub-Test	Homogeneity of Variances Test	Robust Test	ANOVA
			*p* Value of Levene’s Test	*p* Value of Welch Test	
Screening/ Familiarization Tests	Motor Screening (MOT)	MOT Mean latency	0.052		F(4,45)= 0.371, p=0.828
		MOT Mean error	0.203		F(4,45)= 0.507, p=0.731
Visual Memory Test	Paired Associative Learning (PAL)	PAL Total errors (adjusted)	0.097		F(4,45)=0.724, p=0.580
		PAL Total errors (6 shapes, adjusted)	0.516		F(4,45)=1.222, p=0.315
Attention Test	Reaction Time (RTI)	RTI Five-choice movement time	0.298		F(4,45)=0.925, p=0.458
		RTI Five-choice reaction time	0.204		F(4,45)=1.629, p=0.183
	Rapid Visual Information Processing (RVP)	RVP A'	0.041	0.740	F(4,45)=0.417, p=0.796
Executive Function Test	Spatial Working Memory (SWM)	SWM Between errors	0.271		F(4,45)=0.149, p=0.962
		SWM Strategy	0.126		F(4,45)=0.984, p=0.426

One way ANOVA was employed to analyze the five test groups (omeprazole, lansoprazole, rabeprazole, pantoprazole, esomeprazole) at alpha level (<0.05)

### Omeprazole

Compared with baseline, participants receiving omeprazole made more errors in the MOT and PAL tests, took more time for movement in the RTI test, failed to identify correct sequences in RVP, and made more errors in SWM strategy. In the PAL test, the omeprazole group showed a significant (*p <* 0.05) change with 95 % CI and large effect size, whereas in the SWM between error test this group showed a highly significant result (*p =* 0.002) and a large effect size. These findings indicate that omeprazole is one of the major contributors to impaired cognitive function leading to deterioration of visual and episodic memory, new learning, motor and mental response speed, short-term and sustained attention, retention and manipulation of visuospatial information, and strategy development. However, very few significant results were found for omeprazole compared with the control group. Among them, RTI movement time showed a highly significant result (*p <* 0.01) with 95 % CI (43.597 to 311.193) and a large Cohen’s *d* value of 1.313. Overall, the data suggest that omeprazole resulted in both statistically and clinically significant impairment of cognitive performance.

### Lansoprazole

Significant cognitive impairments were observed in the lansoprazole group before and after treatment. Lansoprazole increased the error score in at least one of the MOT, PAL, RTI, and SWM subtests. In addition, the probability of detecting the right sequence in the RVP A′ subtest also decreased significantly (*p <* 0.05). For one of the executive function tests (SWM strategy), lansoprazole produced a more highly significant result (*p =* 0.001), with 95 % CI and a large effect size, compared with any of the other PPIs. These results indicate that lansoprazole not only hampers motor functioning, visual memory, alertness, and attention but also deeply limits the retention of spatial information and the capacity to manipulate remembered memory to develop a strategy and execute a complex task. These findings showed a degree of agreement with previous studies in the mouse brain [21], as they suggested that lansoprazole increases Aβ that might hamper cognition more profoundly than other PPIs do. However, no significant changes (*p >* 0.05) were found in the mean values of all the tests (except PAL) of the volunteers receiving either lansoprazole or placebo.

### Rabeprazole

In the rabeprazole group, MOT and PAL scores did not change significantly either within groups or between groups compared with the control group, suggesting that rabeprazole has little effect on episodic memory or new learning. With regard to the RTI, RVP A′, and SWM tests, however, participants receiving rabeprazole had significantly different scores (*p <* 0.05) compared with their baseline scores. The significant *p* value, 95 % CI, and large effect size for SWM score indicated the negative effect of rabeprazole on the capacity to retain and manipulate spatial memory and planning a task to execute. In addition to within-group significance, the rabeprazole group displayed a significant result (*p <* 0.05) with 95 % CI value compared with the control population.

### Pantoprazole

Compared with other PPIs, the results for pantoprazole are rather elusive. Pantoprazole showed significant changes (*p <* 0.05) in at least one of the MOT, PAL, RTI, and SWM subtests but failed to make notable changes in other subtests. Among them, MOT mean latency, RTI movement time (both within group and between groups), and SWM strategy scores decreased with great significance (*p <* 0.01) and 95 % CI value. In addition, the pantoprazole group showed significant changes in the RVP visual attention test. However, though not conclusively, we might say that participants taking pantoprazole would be at high risk of attention deficit with impaired motor and mental response speeds.

### Esomeprazole

Among the other PPIs, we found little evidence that might support the negative effect of esomeprazole on cognitive performance. The MOT mean error (both within group and between groups), RVP A′, and SWM strategy scores showed significant changes (*p <* 0.05), suggesting that individuals taking esomeprazole would be at high risk of developing difficulties in maintaining sustained attention, retaining and manipulating spatial memory, and planning strategy. Surprisingly, we found no significant effect of esomeprazole on visuospatial memory, new learning, motor and mental response speed, response accuracy, and impulsivity.

Although we found no significant differences among the PPIs by ANOVA, on the basis of paired-samples *t* tests we postulate that omeprazole has the highest impact on cognition and esomeprazole the least. Pantoprazole, lansoprazole, and rabeprazole showed comparatively similar impacts on cognition.

### Summary

Taken together, our findings reveal that different PPIs have some exacerbated effects on cognitive performance. Our findings are in agreement with recent studies suggesting that patients receiving any PPI have a significantly increased risk of developing dementia and AD [17]. However, in this study, we overcame several limitations of previous studies, such as the variety in automated cognitive tests that have been used to assess different domains of cognitive function. Furthermore, we tried to reveal the effect of individual PPIs on cognition. However, several limitations of the present study should also be taken into account. Our study is limited by its short duration and the small number of subjects included. We did not measure the cognitive impact of PPIs after long-term use. Therefore, we analyzed the data from different statistical points of view to reveal the significance of our findings. The presence of additional factors, such as age, sex, apolipoprotein E4 allele, cytochrome P450 2C19 allele, and depression, were not taken into consideration. We investigated the statistical association of PPI use and dementia rather than elucidating the underlying biological mechanism. We do not firmly conclude that all patients receiving chronic PPI therapy will develop AD; rather, these patients may be at high risk of developing some sort of dementia when they are older. AD is a very slow, progressive dementia that takes many years to develop, and many other factors may contribute to its prognosis.

## Conclusions

This study is probably the first of its kind to demonstrate the extent of impairment in different cognitive domains associated with use of different PPIs. Although we estimated the effect only for a short period of time, it is evident that all the PPIs have some exacerbated effects on cognition. In most of the cases, these negative effects may remain unnoticed, but in the long run they may take part in the development of AD. Especially geriatric patients undergoing long-term PPI therapy for chronic acid peptic disorders or taking PPIs for long-term nonsteroidal anti-inflammatory drug therapy are at great risk for developing future AD. As a result, judicious and appropriate use of these medications is imperative to minimizing these risks.

However, to substantiate the robustness of the findings, studies with larger sample sizes and longer follow-up periods are required. Moreover, pharmacogenetic study of PPI-metabolizing enzymes would help to realize the individual variation of cognitive impairment associated with PPI administration. Nevertheless, the present study can be cited as evidence for the effect of PPIs on cognitive performance.

## Abbreviations

AD: Alzheimer’s disease
ANOVA: analysis of variance
ATPase: hydrogen potassium adenosine triphosphatase, proton pump
Aβ: amyloid-β
CANTAB: Cambridge Neuropsychological Test Automated Battery
CI: confidence interval
MOT: Motor Screening Test
PAL: Paired Associates Learning
PPI: proton pump inhibitor
RTI: reaction time
RVP: rapid visual information processing
SWM: spatial working memory
V-ATPase: vacuolar-type ATPase proton pump
